# Tuberous Sclerosis Complex: Early Diagnosis in Infants

**DOI:** 10.15844/pedneurbriefs-32-12

**Published:** 2018-10-17

**Authors:** Stephen L. Nelson, Brittani M. Wild

**Affiliations:** 1 Departments of Pediatrics and Neurology, Tulane University School of Medicine, New Orleans, LA

**Keywords:** Tuberous Sclerosis, TSC, Infants with TSC, Epilepsy, Infantile Spasms

## Abstract

Investigators from the Tuberous Sclerosis Complex (TSC) Autism Center of Excellence Research conducted two concurrent prospective longitudinal studies to analyze the timing and pattern of clinical presenting symptoms of TSC in infants to facilitate earlier diagnosis and treatment in this specific population.

Investigators from the Tuberous Sclerosis Complex (TSC) Autism Center of Excellence Research conducted two concurrent prospective longitudinal studies to analyze the timing and pattern of clinical presenting symptoms of TSC in infants to facilitate earlier diagnosis and treatment in this specific population. There were 130 participants that met diagnostic criteria for TSC. Common initial presenting symptoms were cardiac rhabdomyomas (59%) and hypomelanotic macules (39%), and 85% of the children had either or both. TSC symptoms which led to diagnosis were hypomelanotic macules (94%), tubers and other cortical dysplasias (94%), subpendymal nodules (SENs) (90%), and cardiac rhabdomyomas (82%). All infants had at least one of these features, and 61% had all 4. Prenatally, infants had a higher prevalence of cardiac rhabdomyomas (100% vs. 71%) and a lower prevalence of hypomelanotic macules (87% vs. 98%). Thirty-five percent presented before birth, 41% presented at birth or within the first month of life, and 74% were diagnosed within 30 days of presentation. The mean postnatal diagnosis was 72 days (median 32 days). Neuroimaging findings were present in 115 (94% with tubers or cortical dysplasias, 90% had SENs, and 89% had both). Of the 109 children that underwent genetic testing, pathogenic variants were found in TSC1 (14%) and TSC2 (72%), and 11% had no mutation identified (NMI).

Although seizures are not part of diagnostic criteria for TSC, epilepsy prevalence in TSC has been reported as high as 90%. Mutations in TSC1 resulted in a lower seizure frequency (20%) than TSC2 (87%) or NMI (67%). Infantile spasms (IS) were also more common with TSC2 (68%) compared to NMI (42%) and TSC1 mutations (7%). Furthermore, 15% of infants had seizure onset before or at the time of diagnosis of TSC, suggesting that seizures were the presenting symptom. Seizure prevalence increased over the year after diagnosis (17% at 3 months, 39% at 6 months, and 57% at 1 year). The overall epilepsy frequency was 76% (57% infantile spasms, 55% focal seizures, and 12% other seizure types). Cortical tubers were associated with a high risk for epilepsy (80% vs. 14%). The rate of onset for any seizure type was highest up to 9 months, with infantile spasms risk highest between 3 and 9 months, and focal epilepsy risk highest up to 21 months Earlier seizure onset and higher seizure frequency were associated with worse developmental outcomes. [1]

COMMENTARY. This paper provides important information regarding how to improve early diagnosis of TSC, given that earlier identification and treatment improves developmental outcome. Prenatal ultrasound, neuroimaging, skin examination, and genetic testing are all essential components of this evaluation. The importance of earlier diagnosis is related to the high frequency of epilepsy in this population, and early treatment of seizures is essential [1–3]. Furthermore, TSC is frequently associated with drug resistant epilepsy (DRE), and referral therefore it is essential to aggressively treat to reduce seizure frequency [1–3]. Early referral for epilepsy surgery may be effective in improving seizure control and improving developmental outcome [4,5], and should be considered early for any patient with DRE, including those with TSC.

## Disclosures

The author(s) have declared that no competing interests exist.
